# Monitoring anticoagulation during extracorporeal membrane oxygenation in patients with acute respiratory failure

**DOI:** 10.1186/cc12064

**Published:** 2013-03-19

**Authors:** M Panigada, C Mietto, F Pagan, L Bogno, V Berto, L Gattinoni

**Affiliations:** 1 Fondazione IRCCS Ca' Granda Ospedale Maggiore Policlinico, Milan, Italy; 2 IRCCS Ca' Granda Maggiore Policlinico Hospital Foundation, Milan, Italy

## Introduction

aPTT is a common tool for anticoagulation monitor ing during extracorporeal membrane oxygenation (ECMO). Thromboelasto graphy (TEG) is another available option in this setting.

## Methods

A prospective observational study on 12 consecutive patients during venovenous ECMO. Anticoagulation was provided with unfractioned heparin titrated to an aPTT ratio target of 1.5 to 2. Kaolin-activated TEG (K-TEG) was contemporarily measured but did not guide heparin infusion. Baseline K-TEG reaction time (R) >20 minutes is accepted for anticoagulation but when it exceeds 90 minutes anticoagulation may be too great [1].

## Results

Mean ECMO duration was 9 ± 4 days. A total of 152 K-TEGs were collected. Comparison between aPTT and K-TEG R is reported in Table 1. Four patients (33%) had hemorrhagic complications. Neither aPTT nor K-TEG R were significantly different in patients with hemorrhagic events compared with patients without hemorrhagic events but the latter received a significantly lower total heparin dose (*P *= 0.0097).

**Table 1:** Comparison between aPTT and K-TEG R

	aPTT range (ratio)			
		
K-TEG R range (minutes)	Low (<1.5)	Therapeutic (1.5 to 2)	High (>2)	
**Low (<20)**	13 (9%)	8 (5%)	0	21 (14%)
**Therapeutic (20 to 90)**	10 (7%)	25 (17%)	11 (7%)	46 (30%)
**High (>90)**	15 (10%)	55 (36%)	15 (10%)	85 (56%)
	38 (25%)	88 (58%)	26 (17%)	152 total samples

## Conclusion

Anticoagulation was excessive in more than one-half of the samples according to TEG monitoring, while negligible based on aPTT.

## References

[B1] Oliver WC Semin Cardiothorac Vasc Anesth 2009 13 154 175 10.1177/1089253209347384 19767408

